# Public Space Users’ Soundscape Evaluations in Relation to Their Activities. An Amsterdam-Based Study

**DOI:** 10.3389/fpsyg.2018.01593

**Published:** 2018-08-29

**Authors:** Edda Bild, Karin Pfeffer, Matt Coler, Ori Rubin, Luca Bertolini

**Affiliations:** ^1^Urban Planning Group, Department of Human Geography, Planning and International Development Studies, Faculty of Social and Behavioural Sciences, University of Amsterdam, Amsterdam, Netherlands; ^2^Department of Urban and Regional Planning and Geo-Information Management, Faculty of Geo-Information Science and Earth Observation, University of Twente, Enschede, Netherlands; ^3^University of Groningen, Leeuwarden, Netherlands

**Keywords:** soundscape evaluation, activity, public space, familiarity, expectation, affordance

## Abstract

Understanding the relationship between people and their soundscapes in an urban context of innumerable and diverse sensory stimulations is a difficult endeavor. What public space users hear and how they evaluate it in relation to their performed or intended activities can influence users’ engagement with their spaces as well as their assessment of suitability of public space for their needs or expectations. While the interaction between the auditory experience and activity is a topic gaining momentum in soundscape research, capturing the complexity of this relationship in context remains a multifaceted challenge. In this paper, we address this challenge by researching the user-soundscape relationships in relation to users’ activities. Building on previous soundscape studies, we explore the role and interaction of three potentially influencing factors in users’ soundscape evaluations: level of social interaction of users’ activities, familiarity and expectations, and we employ affordance theory to research the ways in which users bring their soundscapes into use. To this end, we employ a mixed methods design, combining quantitative, qualitative and spatial analyses to analyze how users of three public spaces in Amsterdam evaluate their soundscapes in relation to their activities. We documented the use of an urban park in Amsterdam through non-intrusive behavioral mapping to collect spatial data on observable categories of activities, and integrated our observations with on site questionnaires on ranked soundscape evaluations and free responses detailing users’ evaluations, collected at the same time from park users. One of our key findings is that solitary and socially interactive respondents evaluate their soundscapes differently in relation to their activities, with the latter offering higher suitability and lower disruption ratings than the former; this points to qualitatively different auditory experiences, analyzed further based on users’ open-ended justifications for their evaluations. We provide a methodological contribution (adding to existing soundscape evaluation methodologies), an empirical contribution (providing insight on how users explain their soundscape evaluations in relation to their activities) and a policy and design-related contribution, offering additional insight on a transferable methodology and process that practitioners can employ in their work on the built environment to address the multisensory experience of public spaces.

## Introduction

Research shows that urban sound affects the health and well-being of urbanites in a significant manner, at the same time influencing the use and appreciation of public spaces (Mehta, 2014; Van Kempen et al., 2014). Given this demonstrated importance of sound as part of the urban experience, scientists and practitioners alike have sought to develop strategies to research and influence the relationship between urbanites and their soundscapes, on the one hand to minimize the potential negative effects of sound on urban life, and on the other hand to maximize the opportunities for enjoyment or relaxation that urban sound offers. Whilst extensive attention has been paid to aspects of soundscape evaluation that could potentially feed into effective urban sound policies (Andringa et al., 2013), capturing the complexity of the qualitative urban auditory experience in context (in a real life setting) remains a challenge.

The challenge has both methodological and empirical dimensions, as well as policy and design implications. Strategies focused on how users of various urban spaces evaluate their soundscapes are relatively common in both soundscape research as well as urban policy or practice-related initiatives (see e.g., Axelsson et al., 2010; Booi and van den Berg, 2012; Lercher et al., 2016). However, the conventional methods and tools to study those evaluations are limited in their scope. For example, with regards to public spaces, evaluations are currently mostly collected using questionnaires, largely disregarding other (potentially less invasive) methods that can contribute to a more holistic understanding of the relationship between public space users and their soundscapes, in context. *In situ* methods like field observation (and behavioral mapping) are still rarely used in soundscape research and are currently in the “experimental” stage of implementation with inconsistent results (see Steele et al., 2016; Aletta et al., 2016b; Bild et al., 2018; Lavia et al., 2018, for different approaches). Furthermore, the questionnaires used as tools to gain insight on users’ soundscape evaluations mostly employ categorical-based assessments and rarely include open-ended questions (see Yang and Kang, 2005; Raimbault, 2006, the work in the “Positive Soundscapes Project”^1^, Nielbo et al., 2013; Bild et al., 2018 for examples), thus representing a limited understanding of users’ soundscape evaluations. Finally, these methods minimize or do not adequately account for the role of moderating factors, like activity, in influencing how people evaluate what they hear, despite increasing evidence on activity as a moderating activity for users’ soundscapes (e.g., Aspuru et al., 2011; Bild et al., 2015, 2018; Steffens et al., 2015). The challenge has implications for sound-related urban practice and design initiatives, as it affects the adequate and comprehensive collection and implementation of soundscape knowledge in everyday projects.

In this paper we propose to address these shortcomings in a large-scale, multi-sited urban study based in Amsterdam (Netherlands), where we used a mixed methods approach combining fieldwork observations with questionnaires to capture both reported and “enacted” soundscape evaluations (materialized through public space use). In examining users’ evaluations of their soundscapes in urban public outdoor spaces, we rely on *users’ activities* as a key variable that can influence their evaluations, and, through that, the current and future use of the urban public space (see Nielbo et al., 2013; Steffens et al., 2017; Bild et al., 2018 for comparable approaches). With this in mind, this paper aims to understand the factors that can influence and moderate, both separately and together, how users of three different public spaces evaluate their soundscapes in relation to their on-site activities. Previous soundscape studies indicate three potential factors that can affect the user-soundscape relationship*: the level of social interaction of users’ activities*, users’ (auditory) *expectations* and users’ *familiarity* with the space and with what they hear. To research how the factors interact while influencing users’ evaluations of their soundscapes in relation to their activities, we integrate the concept of *affordance* (Gibson, 1977, 1979) as a conceptual framework for understanding the user-soundscape relationship, focusing on how people bring their soundscapes “into use” in their everyday life (Ingold, 2000), through their activities.

We aim to answer the following two research questions:

(1) To what extent do the level of social interaction of users’ activities, users’ expectations, and users’ familiarity (with the space and with what is heard) influence their soundscape evaluations in relation to their activities, and how are the factors associated?
(2) What are the possible reasons for those associations and how do users describe the factors in relation to their soundscapes?

The scientific work that we build on in this paper is detailed in Section “Background.” The data collection and analysis methods are discussed in Section “Materials and Method” and the findings of the analysis are covered in Section “Results.” We discuss the research and practice-related gaps that we address in detail in the following section and in the concluding discussion (see section “Discussion”); in the latter we also outline the three contributions of this study: empirical, methodological and policy and design-oriented.

## Background

**FIGURE 1:**
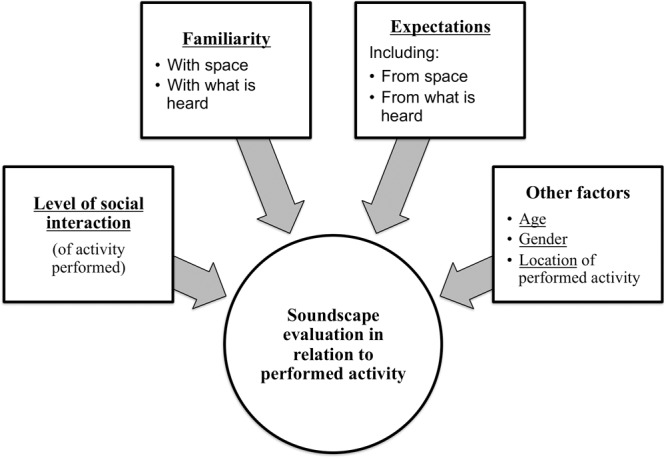
Analytical model to research the user-soundscape relationship in relation to the user’s activity.

The evaluation of soundscapes is at the center of efforts of scientists from disciplines as diverse as psychology or anthropology, particularly of those working at the intersection of theoretical and applied research, as they aim to understand how users of various urban public and private spaces engage with and relate to what they hear, and how that influences the quality of their experience. In this section, we review the scientific literature key to the evaluation of soundscapes and develop the analytical model described below guiding the empirical research (**Figure 1**). First, we discuss studies exploring the role of activity in soundscape evaluations, including the specific effect of *level of social interaction of one’s activities*. Then we review studies researching the role of other factors influencing soundscape evaluations, like one’s previous experience, as it relates to *expectation* and *familiarity*, on these evaluations (discussed below in detail and summarized in the analytical model proposed in **Figure 1**). Finally, we use the concept of *affordance* to understand how users of public spaces bring their soundscapes into use through their engagement with and activities performed in their public spaces (Ingold, 2000; Steenson and Rodger, 2015).

### Soundscape Evaluations and Activity

Scientific efforts have been made to determine soundscape descriptors and indicators that can help explain or predict users’ soundscape evaluations (Nilsson et al., 2007; Jennings and Cain, 2013; Steenson and Rodger, 2015; Herranz-Pascual et al., 2017), with an eye on operationalizing this knowledge and implementing it in sound-related practices. The dominant approach studies evaluations in relation to sound/soundscape quality (see Schulte-Fortkamp and Fiebig, 2016) and integrates aspects of pleasantness (Raimbault, 2006; Axelsson et al., 2010; Can et al., 2016; Herranz-Pascual et al., 2017, *inter alios*) and quietness (Pheasant et al., 2008; Booi and van den Berg, 2012; Bloomfield, 2014; Aspuru et al., 2016), usually in contrast with annoyance (see e.g., Lercher and Schulte-Fortkamp, 2003; Andringa and Lanser, 2013).

While the role of users’ activities as a variable potentially influencing their relationship with their soundscapes has been suggested before (Dubois, 2000; Lercher and Schulte-Fortkamp, 2003), the effective and explicit integration of activity in scientific research with a focus on urban public spaces is still in its incipient, exploratory phase (Aletta et al., 2016b; Bild et al., 2016, 2018; Lavia et al., 2016; Steffens et al., 2017). Most of these research projects arise from more practice-oriented questions, either dealing with specific soundscape interventions with some form of behavioral control in mind (see Lavia et al., 2012, 2016), or emphasizing the role users’ soundscapes play in relaxation or rehabilitation activities or in relation to auditory comfort, both indoors and outdoors (Mzali, 2002; Delepaut, 2009; Cerwén et al., 2016; Filipan et al., 2017). Consequently, many questions remain on how best to define and operationalize *activity* in empirical studies and what methods are suited for researching the relationship between soundscape evaluations and activities in an ecologically valid manner (Guastavino et al., 2005). For example, one laboratory study demonstrated that various soundscape recordings were evaluated as being appropriate^2^ for different imagined activities by participants in a listening experiment (Nielbo et al., 2013); it, however, remains unclear how we can transfer the outcomes from research performed in a laboratory to research performed on-site. We address this issue by furthering the exploration of the aforementioned relationship with a focus on understanding the role activity plays in influencing public space users’ soundscape evaluations. We base part of our inquiry on preliminary studies in the field, showing that *the level of social interaction of users’ activities* has an influence on how users evaluate their soundscapes in relation to their activities (Bild et al., 2018) and a marginal effect on their soundscape descriptions. In other words, to what extent does whether users are alone or with others influence how they evaluate their soundscapes in relation to what they were doing (e.g., talking, reading, sunbathing)?

### Influencing Factor: Expectation

Bruce and Davies (2014) relate soundscape expectations to Truax’s concept of “soundscape competence” (2001), referring to the ability of users of a space to interpret and make sense of what they hear, based on previous experience, and framing soundscape expectations for future situations. Filipan et al. (2017) suggest that the presence or absence of certain *expected* sounds in a context (like a park) can affect users’ evaluations of their soundscapes in terms of, e.g., tranquility. Along similar lines, Bruce et al. (2009, p. 6) argued that a user’s soundscape “becomes an issue when it does not conform to subjects ‘perceived’ sense of normality or interferes with information […] transfer,” thus not conforming to the users’ expectations. The complexity of expectations in relation to one’s experience has been explored extensively apropos music (see e.g., Huron, 2006) and only recently has it been researched explicitly in relation to soundscape (Bruce et al., 2009, 2015; Bruce and Davies, 2014). We build on the conclusions of the latter research avenue, particularly their preliminary findings on the effect of users’ expectations from the space and what they hear on their soundscape evaluations, as well as what users refer to as “expected activities” within the space, as influenced by their soundscapes (Bruce and Davies, 2014).

### Influencing Factor: Familiarity

Familiarity is understood as “how usual or common a stimulus is in the subject’s realm of experience” (Marcell et al., 2000, p. 834), referring to the previous experience of the user with their space, which includes their frequency of use of a space as well as activities performed in the space (Kogan et al., 2017). Particularly for the auditory domain, “familiarity” is one of the three factors that influence the “identifiability” of sounds along with “complexity” (Marcell et al., 2000) and “pleasantness,” as well as one of the three features or perceptual attributes that Axelsson found to be most relevant for users’ evaluations of their soundscapes (third after pleasantness and eventfulness – Axelsson et al., 2010). Axelsson found that variance in familiarity ratings tends to be low for urban respondents sharing a similar cultural framework, thus implying a limited applicability of the feature for design initiatives (Axelsson et al., 2010). We nonetheless consider that users’ reported familiarity both with the space and with what they hear provides valuable insight into users’ evaluations of their soundscapes in relation to their intended or performed activities; familiarity is essential in relation to aspects of expectations, and failure or success to meet them, as it relies on users’ previous knowledge and experience.

### Soundscape and Affordance

Considering the activity-centered approach we take in this paper, we integrate the concept of (auditory) affordances in a public space context. In Gibson’s formulation, affordances are defined as the qualities of an object or an environment that allow for the performance of an activity (Gibson, 1977). Turvey (1992, p. 174) describes an auditory affordance as a way to “provide a description of the environment that was directly relevant to behavior.”. Affordances have been discussed and used previously in auditory research particularly in relation to music, in reference to what music can *afford* to a listener (see DeNora, 2000; Clarke, 2005; Reybrouck, 2012, *inter alios*). There have also been proposals and strategies for integrating the concept in soundscape research (Thibaud, 1998; Pecqueux, 2012; Nielbo et al., 2013; Nielbo, 2015; Steenson and Rodger, 2015) to more accurately address the complexities of user-soundscape relationships and articulate the role that users’ soundscapes play in guiding or informing their public space experiences and uses. We follow Steenson and Rodger’s reading of Gibson in relation to the auditory domain, suggesting that “auditory information is formed relationally, emerging with the situated activity of the agent” (2015, p. 181). We build on the work of Pecqueux (2012), who expands on the idea of affordances elicited by sounds in urban settings (p. 221) and demonstrates the relevance of an activity-centered strategy to researching the urban auditory experience, with implications for design practice. In our approach, we also extend on the idea of “actualization of affordances” (Kyttä, 2002; Stoffregen, 2003), that is, turning possibilities for action into actual activities, focusing on understanding how sounds are brought into use in an urban context (Steenson and Rodger, 2015). We articulate the notion that, by affording users’ activities, users’ soundscapes can enable or impede their activities.

### Proposed Analytical Model

**Figure 1** summarizes the various strands of soundscape research to understand the individual and interaction effect of three factors over users’ soundscape evaluations in relation to their performed activities: (1) the level of the social interaction of users’ performed activities (i.e., solitary vs. socially interactive), (2) expectation (including expectation from the space and from what is heard), and (3) familiarity (with a focus on familiarity with the space and with what is heard). The analytical model informs our mixed methods approach to the evaluation of soundscapes in relation to activity detailed in the next section.

## Materials and Methods

To research to what extent the level of social interaction of users’ activities, users’ expectations, and users’ familiarity (with the space and with what is heard) influence their soundscape evaluations in relation to their activities, and how these factors are associated, we combined quantitative, qualitative and spatial methods in the collection and analysis stages as part of a mixed methods approach (see Creswell and Plano Clark, 2011). Mixed methods approaches are common in soundscape research, as the complexity of people’s urban experiences cannot be fully grasped in mono-method studies (Bloomfield, 2014; Aletta et al., 2016a; Herranz-Pascual et al., 2017; Bild et al., 2018). They are conducive to a more nuanced, situated and integrated exploration of the relationship between users of public spaces and their soundscapes, in context (see also Knigge and Cope, 2006 with respect to the integration of qualitative and quantitative data).

In our research, we relied on a combination of on-site data collection methods, including self-completion questionnaires with randomly selected public space users, and non-participant observation of activities performed in the selected public spaces. The questionnaires included both soundscape evaluations/ratings as well as open-ended questions asking respondents to reflect on their ratings. We collected different types of data suited for both quantitative and qualitative analyses, ultimately contributing to a multi-layered understanding of users’ on-site experience in relation to their activity as follows. The quantitative analysis allowed us to measure potential differences in soundscape ratings between public space users performing different activities and to test the role of various factors in influencing these ratings; the qualitative analysis offered a more nuanced understanding of users’ ratings as well as an in-depth exploration of the reasons behind the aforementioned potential differences between user groups. The non-participant observation of activities was done to situate users’ auditory experiences and their soundscape evaluations in a spatial and behavioral context. In the following sections, we first describe the data collection, including the research design, fieldwork locations and data collection methods, and then elaborate on the data analysis methods.

### Data Collection

We employed a mixed methods research design relying on parallel data gathering. Building on previous pilot studies (Steele et al., 2016; Bild et al., 2018), we combined field observations with on-site questionnaire data collection in a multi-sited field research. We collected data in three public spaces (two large urban public parks and one small urban “plein”/square) over the summer of 2016. 208 self-completion questionnaires were collected with Dutch public space users in similar weather conditions (sunny, warm, and dry), during two data collection sessions per space. Two types of data were collected at the same time: (1) using questionnaires, the ratings and open-ended responses on users’ experiences, and, (2) using field observation, the patterns of occupancy of the public space by solitary and socially interactive users (including the spatial position of users who completed the questionnaire).

#### Fieldwork Locations

**FIGURE 2:**
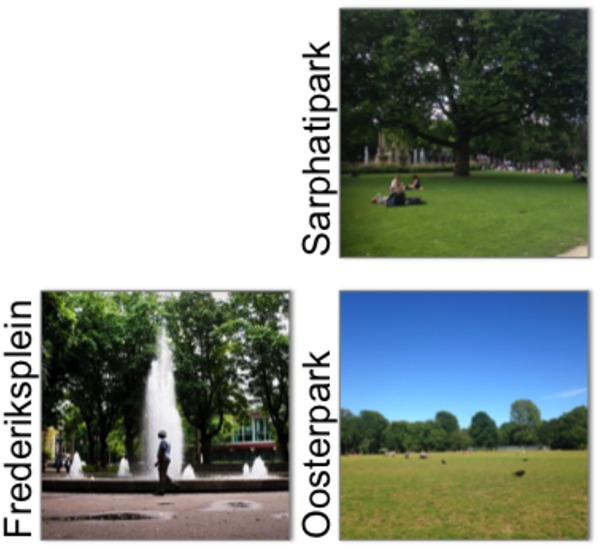
Fieldwork locations. Photo credits: Frederiksplein: Het Parool^3^; Sarphatipark: authors; Oosterpark: authors.

The fieldwork was conducted in various areas of three different locations (**Figure 2**): two traditional urban parks (Oosterpak and Sarphatipark) and one smaller square-park hybrid (Frederiksplein). The spaces are located in central Amsterdam and were selected due to their heavy use for leisure purposes. They represent typical Dutch urban public spaces that can be split in smaller areas bordered by paths and greenery, and are designed with diverse amenities encouraging mixed use and users (see **Table 1** below).

**Table 1:** Fieldwork locations: description and amenities for observed areas.

Location	Description	Amenities
Oosterpark	Large urban park	Large green fields Benches Pond with waterfront green areas Paths Gray/built open area with benches and other sitting possibilities
Sarphatipark	Large urban park	Large green fields Benches Pond with waterfront green areas Paths Water fountain
Frederiksplein	Smaller-sized square-park hybrid Transition space (from center to adjoining neighborhood)	Gray/built open area Benches Paths Water fountain with benches surrounding it Tram tracks cutting through the space

#### Questionnaire Data Collection

##### Questionnaire design

The aim of the questionnaire was to understand whether soundscapes were evaluated as affording users’ activities in a public space context by researching users’ soundscape evaluations in relation to their activities. Questionnaires used in previous research on soundscape evaluations tend to address experiences of spaces in relation to perceptions of pleasantness or eventfulness (e.g., Axelsson et al., 2010; Herranz-Pascual et al., 2017), rarely going in depth on the relationship between use of space and soundscape evaluation. These lines of questioning usually rely on semantic scales and seldom employ additional open-ended questions asking respondents to expand on their evaluations, effectively limiting their applicability in practice (Raimbault, 2006; Nielbo et al., 2013). Current standardized protocols (e.g., the “Soundscape Quality Protocol” – SSQP, Axelsson et al., 2010) might prove insufficient to collect insight useful for both urban researchers aiming to understand the user-soundscape relationship as well as city makers interested in developing spaces with sound in mind, as they would not offer substantial insight into *what* in users’ soundscapes is perceived as disrupting or suitable for their activities or purposes of use of space. We addressed these two challenges in the design of the questionnaire by focusing on users’ soundscape evaluations in relation to their activity and by combining Likert items with open-ended questions in one questionnaire to understand how users reflect on the effect of their soundscapes on their activities and explain potential discrepancies in their evaluations (see **Table 2**).

**Table 2:** Questions (in the order in which they were asked in the questionnaire).

Variable	Question/statement (translation EN)	Question/statement (original NL)	Type of response
Activity (including level of social interaction)	Think back on the activities you perform in this park and **describe** them in as much detail as possible.	Denk terug aan uw activiteiten in dit park vandaag en **beschrijf** deze zo uitgebreid mogelijk.	Open-ended response. Coded for level of social interaction
Disruption	The performance of my activities was disrupted by what I heard.	Het uitvoeren van mijn activiteiten werd verstoord door wat ik hoorde.	Likert item: 1–5 (1, “I completely disagree” to 5, “I completely agree”)
Stimulation	The performance of my activities was stimulated by what I heard.	Het uitvoeren van mijn activiteiten werd gestimuleerd door wat ik hoorde.	Likert item: 1-5
Explanation of disruption or stimulation ratings	In what ways did what you hear disrupt or stimulate the activities you performed?	Op welke manieren werd het uitvoeren van uw activiteiten verstoord dan wel gestimuleerd door wat u hoorde?	Open-ended response
Suitability	What I heard was suitable for the activities that I performed.	Dat wat ik hoorde was toepasselijk voor de activiteiten die ik uitvoerde.	Likert item: 1–5
Expectations	Did you have expectations about this park before you came here? If yes, what were they?	Had u verwachtingen over het park voordat u hier kwam? Zo ja, wat verwachtte u?	Open-ended response
Satisfaction of expectations	In what respect did what you hear match (or not) your expectations?	In hoeverre voldeed wat u hoorde aan uw verwachtingen?	Open-ended response
Familiarity with what is heard	I am familiar with what I heard during the activities I performed.	Ik was bekend met wat ik hoorde tijdens het uitvoeren van mijn activiteiten.	Likert item: 1–5
Familiarity with space (i.e., frequency of use of space)	How often do you visit this park?	Hoe vaak bezoekt u dit park?	Categorical scale: 1–4 (from 1, “Once a week,” to 4, “It is my first time here”)
Age	What is your age?	Wat is uw leeftijd?	Continuous

*Original Dutch and English translation*.

Based on the analytical model outlined in **Figure 1**, we aimed to research the potential influence of three factors on users’ soundscape evaluations in relation to activity, i.e., the level of social interaction of their activity, their familiarity (with what is heard and with the space), and their expectation, and whether these three factors interact for a stronger effect. Additionally, as indicated by literature, we also explored the potential effect of age and gender to influence auditory experiences and potentially the aforementioned evaluations (see e.g., Yang and Kang, 2005).

To understand whether their soundscapes afforded their on-site activities, we asked users to evaluate their soundscapes from three perspectives: in terms of disruption, stimulation and overall suitability; we afterwards asked for detailed explanations of their evaluations (see **Table 2** below). Stimulation is a common term used in relation to soundscapes and particularly in soundscape evaluation usually used as an adjective (Axelsson et al., 2010; Botteldooren et al., 2015), but we use it as an active verb (“to stimulate”). While some authors prefer “to disturb” (and “disturbance”) to convey a similar message (see e.g., Lercher et al., 2016), we selected “to disrupt” as an antonym for “stimulate,” due to its nature as a transitive verb as well as its common use in relation to activity (e.g., Truax, 2001). We did not introduce the concept of “soundscape” in the questionnaire, as we wanted to ensure the statements were phrased in a “natural,” everyday language, allowing respondents to focus on their experience rather than on relating to a new concept.

##### Questionnaire data collection protocol

We approached park users who were usually seated (not in transit), and were willing to engage with the data collector and complete the questionnaire; the questionnaires were completed by native Dutch speakers. Park users were handed clipboards and pens, and were invited to fill out the questionnaires themselves. The data collector offered clarifications when needed. We gathered 188 questionnaires in the three fieldwork locations (Oosterpark: 81 questionnaires, Sarphatipark: 83, Frederiksplein: 24), as part of two data collection sessions per location (one in the weekend and one during the week).

#### Non-participant Observation

To situate the questionnaire data on users’ soundscape evaluations in relation to their activities in a spatial and behavioral context, we also relied on systematic non-participant observation as a fieldwork method, more specifically, behavioral mapping (see e.g., Cosco et al., 2010; Goličnik and Thompson, 2010; Bild et al., 2018). Field observation (Aletta et al., 2016b; Lavia et al., 2016) has been increasingly integrated in urban soundscape research, particularly to document the effects of certain acoustic interventions on the ways in which people engage with and act in their public spaces^3^. Documenting public space use is crucial for on-site studies, as it shows how users relate to and behave in their physical (built) environments and how this relationship can further connect with their soundscape evaluations. By spatially mapping and situating the evaluations of users and their engagement with its amenities and with each other, we can explore how their physical environments and their soundscapes may interact to influence their urban experience in relation to their activities.

**FIGURE 3:**
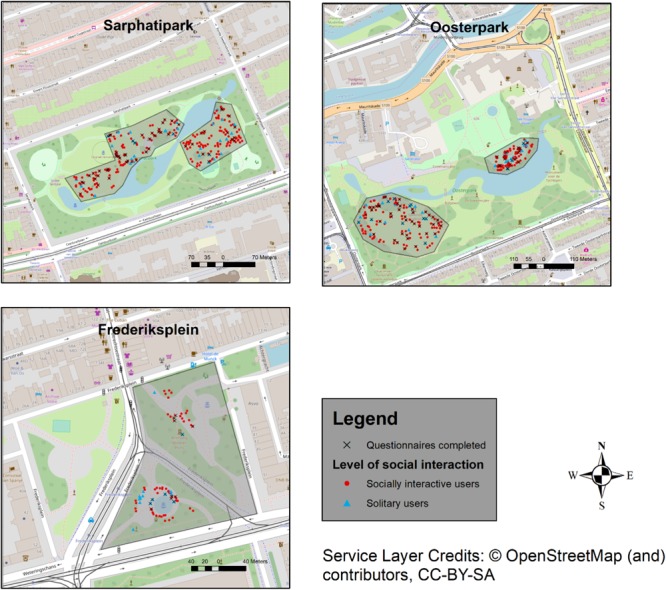
Contextual maps marking the activities observed based on level of social interaction; questionnaires completed in the area also marked. Sarphatipark, Frederiksplein, and Oosterpark.

In this paper, using a behavioral mapping application^4^, we gathered data on the level of social interaction of activities performed by public space users (individual, in pairs or in groups), in parallel with the collection of questionnaires, as part of hour-long sessions throughout the research period. The behavioral mapping resulted in a total of 665 distinct data points, referring to both individual users and users in groups in all three locations, in selected areas of each location. The 665 points include the 188 questionnaire respondents/public space users^5^, and are marked on the resulting behavioral maps (**Figure 3** in section Contextual Maps”).

### Data Analysis

To answer our two research questions, we analyzed the questionnaire data using a sequential approach, first statistically analyzing the responses to the closed-ended questions and afterwards qualitatively analyzing the responses to the open-ended questions. The quantitative analysis served to establish potential patterns in the ways in which solitary and socially interactive respondents evaluate their soundscapes in relation to their activities and the role of, e.g., familiarity as a factor in influencing the evaluation. The qualitative analysis, for which we transcribed and combined the open-ended responses of the questionnaire from all three public spaces, helped to interpret the potential inter-group differences in evaluation and to provide richer, more nuanced knowledge on soundscapes as affordances for respondents’ activities, including exploring the role of expectation as a further factor influencing the evaluation. We created contextual maps to situate users’ questionnaire responses in a spatial and behavioral context.

#### Quantitative Analysis

The variables used in the quantitative analysis are described in **Table 3** below.

**Table 3:** Variables used for quantitative analysis.

**Dependent variables**	
Disruption	5-point Likert item
Stimulation	5-point Likert item
Suitability	5-point Likert item
**Independent variables**	
Level of social interaction	Binary variable (“solitary” and “socially interactive”)
Familiarity with what is heard^∗^	Ordinal variable (“low and medium familiarity,” “high familiarity,” “very high familiarity”)
Frequency of use of public space	Ordinal variable (“this is my first visit,” “a few times per year,” “at least once a month,” “at least once a week”)
Location	Categorical variable; three distinct locations (“Sarphatipark,” “Oosterpark,” “Frederiksplein”)
Age^∗∗^	Binary variable: “35 or younger” and “older than 35”
Gender	Binary variable: “male,” and “female”

*^∗^The original five categories were collapsed in three for group comparison: “low and medium familiarity” (including “very low,” “low,” and “medium familiarity”), “high familiarity” and “very high familiarity.” ^∗∗^The original continuous “age” variable was collapsed in two for group comparison*.

The three dependent variables in the analysis – disruption, stimulation and suitability were measured on a 5-point ordinal scale and in our data were non-normally distributed, so we relied on two non-parametric tests for our analysis (Ruxton, 2006). First, using the Kruskal–Wallis test, we tested whether there are statistically significant differences between the categories of the independent variables on each of the three soundscape evaluations. Second, we applied the Mann–Whitney *U* test to investigate whether soundscape evaluations differed significantly between activity types (solitary or socially interactive) according to frequency of use, familiarity with what is heard, location, age and gender. We considered relationships with *p*< 0.05 as statistically significant. We also discussed cases where *p*< 0.1 to indicate trends in the data, given the limited sample and number of variables we had at our disposal. The quantitative analysis was performed with the help of statistics software (SPSS version 19).

#### Qualitative Analysis

We performed an in-depth analysis of responses to the open-ended questions that respondents provided when asked to explain how their soundscape stimulated or disrupted their activities (if at all), focusing on *what was disrupting/stimulating* (cause) and *what was disrupted/stimulated* (effect). We also analyzed how respondents articulated their (auditory) expectations and whether they were met during their time in the space. Our thematic coding approach was inspired by previous work on soundscape and place expectations (Bruce and Davies, 2014), focusing on respondents’ expectations from the space itself, their auditory expectations (namely expected sounds), what they expected to experience in the space as well as expectations from others present in the space. We contrasted the answers of respondents performing solitary activities with those performing socially interactive activities.

#### Contextual Maps

We visualized the data collected through behavioral mapping for the three fieldwork locations using GIS-based methods to situate the data on soundscape evaluation in a spatial setting. The resulting maps show the spatial distribution of questionnaires and are accompanied by an overview of patterns of occupancy for each observed location, in relation to the level of social interaction of users’ activities, illustrating the social interaction context within which the questionnaire responses were collected.

## Results

### Contextual Maps

We begin with the analysis of the maps resulting from the behavioral mapping process (**Figure 3**) as they play a descriptive role, that is, to illustrate the larger context in which the questionnaires were filled out in terms of patterns of use based on the level of social interaction of the activities performed. The maps for each public space are an aggregation of the data collected during the two sessions per space and visualize the use of space exclusively in the areas where the behavioral mapping was carried out (marked with light gray in the resulting maps); the other areas have not been observed due to practical reasons, yet they were also consistently frequented by users.

The maps clearly show that socially interactive users are dominant in the space, throughout all three locations. The main observed physical factors influencing the distribution of use and subsequent concentration of users were: the surface materials (i.e., pavement or grass), presence/absence of shade (influenced by trees and other greenery), location and presence of conventional seating amenities (i.e., benches) or other elements that could used as seating amenities (e.g., other built structures), points of attraction (e.g., water fountains), proximity to bodies of water (i.e., ponds), and proximity to foot/bicycle paths.

The less dense, more spread out occupancy of the large open area in, e.g., the Western part of Oosterpark or all of Sarphatipark was influenced by the existence of conventional seating amenities mainly along the foot/bicycle paths, with large open grass fields in between. We observed the clustering of users both in Eastern Oosterpark and throughout Frederiksplein. This could be due to the lack of grass where users could sit on and the dominance of various seating amenities (users, mostly socially interactive, also sat on the side of the fountain in Frederiksplein, and on round, elevated built structures in Oosterpark). The users closest to the body of water in Easter Oosterpark, largely performing socially interactive activities, were facing the water while sitting on grass, whereas solitary users mostly faced the water from a larger distance, while sitting on benches. The clear dominance and clustering of socially interactive users in the NE section of Sarphatipark was due to three separate birthday celebrations taking place at the same time, bringing together large groups of users.

The location of the completed questionnaires document in **Figure 3** demonstrates that the sample of users approached to complete our questionnaires is representative for the distribution of users in space in the timeframe and the locations where we conducted our research, with socially interactive users dominant across spaces, usually occupying the larger grass fields (generally in the sun), and solitary users equally distributed between the open fields and seating amenities closer to the paths (the latter generally in the shade).

### Quantitative Results: Statistical Analyses of Soundscape Evaluations

The sample distribution according to the main variables (**Table 4**) shows that, for the dependent variables, the vast majority of respondents (86%) evaluated their soundscapes as having low or very low disruption values, while no respondent evaluated them as being very disrupting. The sample was split rather evenly for stimulation ratings (low, medium and high stimulation), with around 30% of respondents each. The majority of respondents (67%) evaluated their soundscapes as highly or very highly suitable for their activities. Most of the respondents were participating in socially interactive activities. The sample was divided rather evenly also by frequency, with 47% visiting the locations at least once a month. The vast majority of respondents (90%) stated to be highly or very highly familiar with their soundscapes. 76% were 35 or younger and a slight majority of the sample identified as female.

**Table 4:** Distribution of valid responses by variable used in quantitative analyses.

Variable	Values	*N* (%)
Level of social interaction	Socially interactive	127 (67.6%)
	Solitary	61 (32.4%)
Frequency of use of space	This is my first visit	30 (16%)
	A few times per year	70 (37.2%)
	At least once a month	58 (30.9%)
	At least once a week	30 (16%)
Familiarity with what is heard	Low and medium familiarity	18 (9.6%)
	High familiarity	57 (30.3%)
	Very high familiarity	113 (60.1%)
Location	Sarphatipark	83 (44.1%)
	Oosterpark	81 (43.1%)
	Frederiksplein	24 (12.8%)
Age	35 or younger	143 (76.1%)
	Older than 35	45 (23.9%)
Gender	Male	85 (45.2%)
	Female	103 (54.8%)
Disruption	Very low disruption	111 (59%)
	Low disruption	58 (30.9%)
	Medium disruption	11 (5.9%)
	High disruption	8 (4.3%)
	Very high disruption	0
Stimulation	Very low stimulation	29 (15.4%)
	Low stimulation	29 (15.4%)
	Medium stimulation	68 (36.2%)
	High stimulation	47 (25%)
	Very high stimulation	15 (8%)
Suitability	Very low suitability	11 (5.9%)
	Low suitability	11 (5.9%)
	Medium suitability	41 (21.8%)
	High suitability	80 (42.6%)
	Very high suitability	45 (23.9%)

*(N = 188).*

**FIGURE 4:**
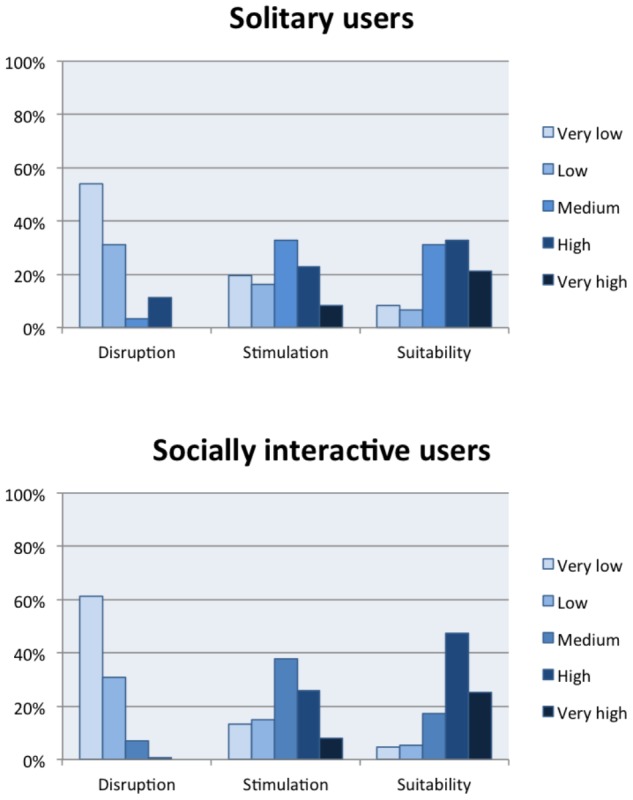
Distribution of soundscape evaluations in relation to respondents’ level of social interaction.

The distribution of soundscape ratings split by level of social interaction is presented in **Figure 4**. For disruption, a larger share of solitary respondents evaluated their soundscapes as having very high levels of disruption than socially interactive users; 11% of solitary respondents evaluated their soundscapes as highly or very highly disruptive, compared to 1% of socially interactive respondents. For stimulation, a larger share of solitary users evaluated their soundscape as having very low or low levels of stimulation: 36% compared to 28% of socially interactive users. Finally, for suitability, a smaller share of solitary respondents evaluated their soundscapes as highly or very highly suitable (54% of respondents compared to 72% of socially interactive respondents).

The Kruskal–Wallis test was conducted to evaluate the differences between the categories of the five independent variables (level of social interaction, frequency of use of space, familiarity with what is heard, location and age) on the three soundscape evaluations (disruption, stimulation and suitability). The results (**Table 5**) showed that there was a significant difference in suitability rating for all independent variables, albeit a weak significance for level of social interaction; there was also a significant difference in disruption ratings between the three locations. These differences demonstrate the relevance of the independent variables in influencing the extent to which respondents’ soundscapes are perceived to afford/be suitable for their on-site activities. Overall, the tests showed that the independent variables included in this research are related mainly with suitability ratings and minimally disruption ratings. The independent variables do not significantly relate with stimulation ratings. This suggests that “suitability” is the clearest construct for respondents to grasp, while “stimulation,” and to an extent “disruption,” are somewhat more challenging to assess.

**Table 5:** Results for the Kruskal–Wallis test to compare the soundscape evaluations between categories of independent variables.

Variables	Disruption	Stimulation	Suitability
Level of social interaction	1.668	0.739	3.523^∗^
Frequency of use of space	2.270	5.849	13.066^∗∗^
Familiarity with what is heard	4.027	2.222	11.230^∗∗^
Location	17.897^∗∗^	2.543	9.474^∗∗^
Age	0.416	0.547	4.547^∗∗^

*Chi-square values reported. P-value significance: ^∗∗^ for p < 0.05, ^∗^ for p < 0.1 (trend of significance). N = 188.*

To further understand the relationships identified above, we used the Mann–Whitney *U* test to calculate whether soundscape ratings, grouped by the level of social interaction of respondents’ activities, differ among categories of the independent variables (**Table 6**).

**Table 6:** Results of the Mann–Whitney *U* test: comparison of soundscape evaluations between users according to level of social interaction.

Variable	Values	Disr. SI (mean rank)	Disr. Sol (mean rank)	Disr. (sig.) Mann–Whitney *U*	Stim. SI (mean rank)	Stim. Sol (mean rank)	Stim. (sig.) Mann–Whitney *U*	Suitability SI (mean rank)	Suitability Sol (mean rank)	Suitability (sig) Mann–Whitney *U*
Frequency of use of space	This is my first visit	14.39	19.14	55.000	17.50	8.93	34.500^∗∗^	17.50	8.93	34.500^∗∗^
	A few times per year	34.69	33.97	388.500	35.95	33.97	407.500	36.85	30.94	359.000
	At least once a month	28.13	31.59	354.500	32.26	25.30	306.000	30.37	28.17	372.000
	At least once a week	14.93	16.07	104.000	12.93	18.07	74.000^∗^	17.23	13.77	86.500
Familiarity with what is heard	Low and medium familiarity	9.58	9.33	35.000	9.92	8.67	31.000	10.29	7.92	26.500
	High familiarity	26.98	35.85	197.000^∗^	29.41	27.62	268.000	30.00	25.62	242.000
	Very high familiarity	55.18	60.07	1362.000	58.01	55.29	1419.000	60.96	50.30	1209.500
Location	Sarphatipark	39.10	51.15	447.000^∗∗^	44.15	35.23	494.500	45.36	31.43	418.500^∗∗^
	Oosterpark	41.64	39.79	708.000	40.12	42.66	695.500	41.88	39.34	695.500
	Frederiksplein	12.68	12.35	69.500	13.18	11.92	64.000	12.23	12.73	68.500
Age	35 or younger	70.93	74.86	1916.500	72.02	71.94	2025.500	75.52	62.62	1662.000
	Older than 35	20.22	25.91	189.000^∗^	26.30	19.55	177.000^∗^	25.83	20.05	188.000
Gender	Male	41.39	45.09	810.500	44.86	40.58	798.500	45.40	39.89	773.000
	Female	50.46	57.08	826.000	52.51	50.33	908.000	54.41	44.08	758.000

*Mean rank and Mann–Whitney U values reported (the higher the mean rank, the higher the rating). Dependent variables: disruption (“disr.”), stimulation (“stim.”), and suitability (“suit.”). Level of social interaction: solitary (“Sol”) and socially interactive (“SI”). P-value significance (“sig.”): ^∗∗^ for p < 0.05, ^∗^ for p < 0.1 (trend of significance). N = 188.*

The Mann–Whitney *U* test indicated that for those visiting the locations for the first time, socially interactive respondents have significantly higher stimulation ratings than solitary respondents (*U* = 34.500, *p* < 0.05), and higher suitability ratings, albeit with a weak significance (*U* = 34.500, *p* < 0.05). A possible explanation could be that the locations researched here are more geared toward group activities. Groups were especially dominant in those spaces on sunny days, usually engaged in various – likely audible – interactive activities throughout the observed areas (as seen in the contextual maps in **Figure 3**). Also among respondents who visit at least weekly, evaluations of stimulation were higher – although weakly significant – for socially interactive respondents than for solitary respondents (*U* = 74.000, *p* < 0.1).

For respondents with high familiarity ratings, there is a weakly significant difference between socially interactive and solitary respondents, with the former having lower disruption ratings than the latter (*U* = 197.000, *p* < 0.1).

In the particular case of Sarphatipark, socially interactive respondents have significantly lower disruption and significantly higher suitability ratings than solitary respondents (*U* = 447.000, *p* < 0.05, and *U* = 418.5000, *p* < 0.05, respectively). This suggests that Sarphatipark is uniquely perceived as affording socially interactive activities rather than solitary ones in a significant manner, when compared to the other two locations.

For users older than 35, socially interactive respondents have lower disruption and higher stimulation ratings than solitary respondents, albeit weakly significant (*U* = 189.000, *p* < 0.1, and *U* = 177.000, *p* < 0.1, respectively). Finally, no significant differences between socially interactive users and solitary ones were found for males and females.

### Qualitative Results

The quantitative analysis partially confirmed our literature-driven expectations on the role of age, the level of social interaction of respondents’ activities and of respondents’ familiarity, both with the space and with what they hear, on their soundscape ratings in relation to their activity. The location in which the research was conducted was also identified as having an effect on soundscape ratings, particularly for Sarphatipark. The findings provided little detail on the respondents’ experience that could guide, for example, design interventions, e.g., what they find disrupting or stimulating or what their expectations were from their space and their soundscapes, thus leaving much to speculation. To address this, we relied on qualitative insights from an in-depth analysis of respondents’ explanations of their disruption and stimulation ratings, as well as their expectations, to better understand what specifically in their space and their soundscapes affords (or discourages) their activities. We first grouped the responses of all three spaces together, and categorized respondents’ descriptions of their expectations according to: the type of space they were expecting to find, its amenities, what they expected to hear and how they expected others to use the space. Considering that Sarphatipark stood out in the quantitative analysis as a space evaluated as particularly affording of respondents’ activities, we investigated whether the responses in the park differed from those in the other two fieldwork locations. However, no particular differences were observed, so below we report only on the aggregated data from the three spaces.

#### Explanation of Disruption and Stimulation Ratings

Respondents described *how* their soundscapes disrupted and/or stimulated their activities, with a particular emphasis on *what* in their soundscapes they considered to be disrupting or stimulating.

##### Disruption

The main source of disruption was, for both solitary and socially interactive respondents, the sounds of others in the space, especially the sounds of loud conversations and of children crying; surprisingly, the sounds of traffic (and public transportation) were mentioned only in passing as a source of disruption, the focus remaining on other public space users and their sound-producing activities. Solitary respondents also tended to cite more holistic reasons for their disturbance (e.g., “city sounds,” “all sounds,” “racket”^6^) than socially interactive respondents.

Both solitary and socially interactive respondents focused on the disturbing/distracting effect that some sounds had over their own activity: in the case of solitary respondents, what they heard disturbed their thought process or their ability to unwind, whereas for socially interactive respondents, their conversation was interrupted or they had to adjust their speaking levels to be able to understand each other.

##### Stimulation

While for sources of disruption, there was quite some consensus on which sources are considered disrupting (see above) and a relatively small number of sounds were listed, there was a comparatively larger array of sources of stimulation mentioned by both categories of users. Socially interactive respondents stood out by listing comparatively more aspects of their auditory experience that they considered stimulating, including not only sounds but also using more holistic descriptions like “coziness” (“gezelligheid”). The sources of stimulation were, to relatively equal extents, nature-related sounds (i.e., fountain, birds, water, with socially interactive respondents putting an emphasis on the sound of wind through the leaves of trees) and human activity-related sounds.

Both solitary and socially interactive respondents focused on how what they heard stimulated the “atmosphere” in their space and the effect it had over users, particularly in relation to a relaxing^7^ effect or to a “holiday feeling”: “the buzz/murmur contributes to a pleasant atmosphere”^8^. Interestingly enough, solitary respondents focused particularly on how what they heard stimulated hypothetical conversations (“if I hear other people talk, it is also easier for me to talk”^9^) or doing what they wanted (“I’m stimulated to do what I like”^10^), e.g., fall asleep (“calming sounds allow me to fall asleep”^11^). Comparatively, socially interactive respondents further emphasized the importance of the presence of others for coziness and cheerfulness: “The fact that you can hear life around you makes it pleasant and cozy. In either way, it makes [this] pleasant and cozy”.^12^

Solitary respondents focused more on the effect of what they heard had on their intended or current activities, whereas socially interactive respondents were more embedded in and engaged with their soundscapes, emphasizing not only the quiet dimension of their experience, but also the dynamism generated by the presence of others.

One socially interactive respondent (offering a low disruption rating and a high stimulation rating) summarized the complexity of their relationship with their soundscape: “music offers an atmosphere, so does the water and people. The tram is a bit disturbing but it is allowed here in the city”^13^.

Not all respondents that offered explanations to their disruption or stimulation ratings identified particular sounds that affected their evaluation. Some respondents focused only on one or two disrupting sounds, stating that “the rest” is neither stimulating nor disruptive. Others stated that some sounds were “distracting,” but that in general they were neither stimulated nor stimulated by what they heard; a sub-group of respondents stated that they were too focused on their activity to be aware of their soundscape: “I was very busy with my own activity so I was not very aware of the ambient sound”^14^ .

#### Users’ Expectations From Their On-Site Experiences

A count of occurrences showed that the majority of respondents reported that their expectations were met in all three spaces during their activities; however, only a slight majority of solitary respondents felt their expectations were met, compared to slightly over three quarters of socially interactive respondents. The subtle differences in expectations between solitary and socially interactive respondents indicate slightly different auditory experiences for those who use the public spaces alone or with others. As indicated by Bruce and Davies (2014), public space users tend to expect a limited number of sounds in an urban environment, especially for leisure-related uses and in relation to urban parks. Both categories of respondents expected the sound of fountain and water (due to two of the three public spaces being designed with large water fountains around which users tended to cluster, as shown in “Contextual Maps,” and visualized in **Figure 3**) as well as “city sounds,” However, socially interactive respondents also expected to hear the sounds of birds, which solitary respondents did not mention in their responses: “quiet environment with a fountain and birds”^15^. Furthermore, only socially interactive respondents stated they expected to hear the sounds of people and traffic-related sounds: “many people because of the nice weather. Tram + car also expected because we are close to the road. Oosterpark is not so big”^16^.

Solitary respondents were more likely than socially interactive respondents to expect *quietness* first, with *crowdedness* mentioned second; the latter placed *crowdedness* first in their list of expectations, followed by *quietness* and, equally important, *atmosphere* (whatever it entailed for respondents, usually in relation to *coziness*). Not surprisingly, in relation to the expected behavior of *others* in the public space, both groups of users expected the presence of others; however, while solitary respondents referred only marginally to the expected behavior of others, a large proportion of socially interactive respondents specifically mentioned they expected the presence of others when they decided to use the public spaces. Furthermore, they emphasized the expected level of interaction and dynamism of the activities that *others* would be performing: “crowdedness, many groups of people, young men playing football”^17^.

Finally, in relation to expectations from the public spaces themselves, both categories of respondents stated that they expected a *city park* (“a park, just like any other park in Amsterdam”^18^), which comes with its assumptions in terms of patterns of use (shown in **Figure 3**) and, of course, audible sounds. This is particularly interesting for the case of Frederiksplein, not a traditional large urban park but rather a small urban square – park hybrid (a “plein”).

Despite the variety in expectations, a majority of both solitary and socially interactive respondents stated their expectations were mostly or fully met, with some respondents explaining that their expectations were influenced by their previous uses of the park: “I was here before so I knew what I could expect”^19^.

## Discussion

This paper employed a mixed methods approach to study the user-soundscape relationship in a public space context, with an emphasis on users’ activities; we further investigated how the level of social interaction of users’ activities, individually or interacting with other factors, influence users’ evaluations of their soundscapes, following the analytical model introduced earlier. We thus sought to demonstrate the relevance of considering the relationship between activity and soundscape evaluations when designing spaces for specific *uses* and interactions, rather than exclusively for generic goals like restoration. Through a mixed methods approach, we tested a number of factors that soundscape literature suggested to be likely to influence the user-soundscape relationship in a specific context, given the users’ activities. We framed our research questions in relation to affordance theory, which helped to explain how public space users refer and evaluate the relationship between what they hear and what they do, i.e., do they evaluate their soundscapes as disruptive, stimulating or overall suitable for their activity.

We make three contributions to help address the challenge detailed in the introduction, and discuss each one below:

(1) A methodological contribution, adding to existing soundscape evaluation methodologies, reflecting at the same time on the limitations and ways of improving current methods,
(2) An empirical contribution, providing insight on how users explain their soundscape evaluations in relation to their activities and building on previous use of affordance theory to further the idea of a strong relationship between users’ soundscape evaluations and their activities
(3) A policy and design-related contribution, offering additional insight on a transferable methodology/process that practitioners can employ in their work on the built environment.

### Methodological Contribution and Reflection

We used a mixed methods approach for a multi-layered analysis of the user-soundscape relationship in a public space setting, by (1) integrating users’ activities as a key variable framing evaluations, (2) exploring the individual and interaction effect of additional factors on this relationship, (3) combining Likert items with open-ended responses where users can explain their soundscape ratings, and (4) combining questionnaires with on-site behavioral mapping to situate users’ soundscape evaluations in a public space context.

The behavioral mapping was used to integrate a spatial dimension and to situate the data collected through questionnaires, not only in a physical environment, but also in a behavioral setting of *others* performing activities, that can offer additional insight into user evaluations and that cannot sufficiently be grasped via questionnaires alone. The quantitative method was used to collect categorical data on public space users’ evaluations of their soundscapes in order to compare the ratings between users engaged in activities with different levels of social interaction, as well as across various factors that might influence the user-soundscape relationship. The qualitative analysis was used to offer more depth to the statistical findings; as the quantitative findings show a similar trend in soundscape ratings, the qualitative insight helped to understand the subtle differences in ratings and offer an interpretation of the findings. As people continue using the public spaces despite some (albeit low) level of reported disruption, only an in-depth approach could allow researchers and practitioners to understand, e.g., what are the sources of disruption and what makes users apparently accept them. Open-ended responses were encouraged through open-ended questions, which meant offering users the space to reflect on their experience and their subsequent evaluations. While analyzing such responses is time-consuming, it allows researchers and practitioners alike to make sure that they understand what the users of spaces are experiencing, focusing on and, ultimately, evaluating; simply asking users if they “like” what they hear in a space or if they find it “pleasant” is insufficient, as responses to such questions can potentially lead the data collector (designer, planner, researcher, etc.) to resort to a top-down interpretation of *what* the users evaluated. The knowledge collected through open-ended responses was thus essential in understanding what users focused on and referred to in their evaluation, as well as grasping the specific aspects in their experience that disrupted or stimulated their use of spaces, thus allowing for an exploration of sounds and soundscapes as affordances for users’ activities on site.

In this paper, we provide practitioners and researchers with an example of an insightful research *process* and with methods and tools to observe, ask and engage with users (actual or potential) of public spaces in relation to their multisensory experience. The methodology we put forward can also be used to research and document other aspects of the built environment, without being restricted to the auditory experience. We thus do not put forward a one-size-fits-all model, but rather a qualitative user-centered process that must be adapted to the specific and unique needs of each case, but that can provide a wealth of knowledge on *what* disrupts or stimulates users’ activities in a public space. However, one minor limitation of this study method is that single Likert items were the variables analyzed (disruption, suitability, and stimulation); a future approach would be to substitute these with validated multi-item Likert scales as variables instead. For example, considering that suitability is indicated as the most useful/robust rating for users’ soundscapes, it would be worth it in future studies to formulate a “suitability scale” based on multiple items, which may incorporate disruption and stimulation as well as other variables as the ones explored in this paper.

While time consuming and heavily reliant on users’ willingness to participate, the methodology is nevertheless valuable for understanding what types of activities users’ soundscapes and physical environments afford. A limitation of our mixed methods approach is that it questions and observes current users of mostly green parks, that are therefore less likely to have negative evaluations of their environment and their experience (as seen in the largely positive soundscape evaluations of both solitary and socially interactive respondents). Furthermore, asking users to reflect on what they hear through questionnaires encourages them to actively focus on their soundscapes, which results in responses that might not fully reflect their on-site everyday auditory experiences. This shows the need for further improving our methodology to elicit auditory knowledge in more creative, but systematic ways. The study described here is a first effort to research this topic on site and the questions raised by the results of our data can be used as opportunities for guiding or improving future research. For example, further attention could be paid to developing additional protocols to analyze the responses to the open-ended question on activity or on testing hypotheses on specific auditory affordances that soundscapes “create” for public space users, in terms directly relevant to users’ activities or behavior. Furthermore, given the responses of, e.g., first-time solitary respondents, who evaluated their soundscapes as less suitable for their activities than first-time socially interactive respondents, an additional line of inquiry could be focused on their likeliness to return in the future to the public space in the future (or would prefer a different space). This could provide insight into whether there are aspects of their auditory experience in that particular location that have failed to meet the needs of various solitary respondents on multiple occasions. Finally, to better benefit from behavioral mapping as a method, more complex data could be collected on space users and uses, for example more detailed insight on the type of social interaction they are engaged in, e.g., families with children, pairs, and more sophisticated spatial statistics could be employed to analyze the relationship between characteristics of the space, its patterns of use and users’ soundscape evaluations.

### Empirical Contribution

The behavioral mapping showed the patterns of use of the three fieldwork locations by both solitary and socially interactive users during the research period, as a context in which the questionnaire respondents provided their soundscape evaluations. The quantitative analysis indicated that the level of social interaction of users’ activities had an association with their suitability ratings, albeit weakly significant. It also showed that familiarity levels, both with what was heard and with the space (frequency of use of space) differed significantly for suitability ratings. The experience of first time visitors was of particular interest, as there was a significant difference between stimulation and suitability ratings for solitary and socially interactive respondents (with the latter being having higher ratings than the former for both ratings). For frequent users, a weakly significant association was shown in relation to stimulation ratings, with socially interactive users having higher stimulating evaluations than solitary users. Location also had a statistically significant association with disruption and suitability ratings, particularly for Sarphatipark, where solitary respondents reported significantly lower suitability and higher disruption ratings than socially interactive respondents. This difference in ratings also holds true for users older than 35 across locations for disruption and stimulation ratings, for whom the differences between solitary and socially interactive are weakly significant.

The qualitative analysis confirmed that solitary and socially interactive respondents differed slightly both in terms of sources of disruption and stimulation, as well as in the particular expectations (auditory and otherwise) from their experience. The sounds of people were considered as the main source of both disruption and stimulation for both groups; while conversations and the sounds of others in general were referred to as stimulating, *loud* conversations and children crying were disrupting. Surprisingly, the sounds of traffic were not mentioned as a main source of disruption; unsurprisingly, “natural” sounds were mentioned as a main source of stimulation (with only socially interactive respondents mentioning birds among stimulating sources). While solitary respondents were more likely to include holistic sounds (e.g., “city sounds”) among sources of disruption, socially interactive respondents were more likely to include such sounds among sources of stimulation, thus affording their activities (e.g., “atmosphere,” “buzz/murmur”). In terms of expectations, both solitary and socially interactive respondents reported that their expectations were largely met, which explains the soundscape ratings in relation to their activities reported on in the previous section, i.e., overall low disruption ratings and high stimulation and suitability ratings. Socially interactive respondents tended to focus not only on the presence of others, but also on their activities as well as the others’ levels of social interaction. They were also more likely to emphasize the importance of the general atmosphere/ambiance in their expectations, whereas solitary respondents focused on their expectations in relation to quietness. In terms of sounds expected, socially interactive respondents tended to expect a larger variety of “natural sounds” (including birds, wind in the trees, etc.), as well as more traffic and street-life related sounds (e.g., cars, tram, “the street”). The presence of others, not only as a source of disruption but rather as an *affordance* that both helps in the “creation of atmosphere” and encourages one’s own engagement with the space is thus essential when discussing/addressing auditory concerns in relation to public space use.

### Policy/Design Implications

The policy and design implications are twofold, based on the empirical findings, as well as our methodological approach. On the one hand, the empirical insights demonstrate the added value of considering users’ soundscapes in relation to their activities (with a focus on whether the activities performed are solitary or socially interactive) when considering new policy or design initiatives; it also showed the potential of including the analytical framework developed in the background section to help unpacking the complexity of the auditory experience. For example, tools like questionnaires commonly used by various practitioners should include activity questions in soundscape-related queries, such as asking users about their activity and whether they were by themselves or with others at the time of the completion of, for example, noise exposure surveys.

On the other hand, the methodological approach described in this paper, and the resulting research process described above can be used by policy makers and designers to gain contextual insights in users’ experiences. For example, integrating open-ended questions in current questionnaires for soundscape evaluations can help with verifying the suitability or relevance of commonly (and uncritically) used terms like “annoyance” or “pleasantness” by accessing the everyday sound-related vocabulary of urbanites, which can in turn feed into and help adjust existing tools used by local, regional and national authorities.

Overall, in this paper, we provided both empirical and methodological insights that researchers and practitioners alike can adjust and employ in their own investigations of urban auditory complexity to contribute to the creation of spaces that afford a large array of activities. For future research and practice, it would be interesting to explore whether using richer descriptions or evaluations brings designers and policy makers to different kinds of interventions.

## Ethics Statement

This study was carried out in accordance with the guidelines outlined by the Ethics Committee (AIEC) of the University of Amsterdam. The participation in the study was voluntary and the responses were fully anonymized; subjects were informed that, if they completed the questionnaires, their answers would be used in a scientific study. By completing the written questionnaires, they confirmed that they provided consent to participate in the research.

## Author Contributions

EB performed the data collection and analyses and wrote the majority of the paper. KP, MC, and LB provided extensive comments and textual edits on previous versions of the manuscript. OR contributed to the quantitative analysis in this manuscript. The doctoral project this research is part of was initiated by MC, as part of INCAS, and further elaborated jointly by EB, KP, MC, and LB.

## Conflict of Interest Statement

The authors declare that the research was conducted in the absence of any commercial or financial relationships that could be construed as a potential conflict of interest.

## Footnotes

1 For a review of papers published in this project, check the project URL at: https://www.salford.ac.uk/research/sirc/research-groups/acoustics/psychoacoustics/positive-soundscapes-project [Accessed May 2nd 2018].

2 The term “appropriateness” has been used to measure users’ evaluation of their soundscapes in relation to their settings, defined either as their performed or imagined activity – appropriateness for activity (Nielbo et al., 2013; Bild et al., 2018) or, more commonly, the geographical setting they find themselves in – appropriateness in/for “a place” (Lavia et al., 2012; Aletta et al., 2016a). In this paper, we focus on the former understanding of the term.

3 For another application of this method as part of a mixed methods approach, see Steele et al. (2016).

4 The method has been tested and implemented in two previous smaller scale studies (viz. Bild et al., 2018, for a detailed explanation of the behavioral mapping method and tools; see also Steele et al., 2016).

5 We hereinafter refer to the public space users who completed our questionnaire as “respondents.”

6 Original in Dutch: “lawaai.”

7 Original in Dutch: “rustgevend.”

8 Original in Dutch: “het geroezemoes draagt bij aan gemoedelijke sfeer.”

9 Original in Dutch: “als ik andere mensen hoor praten, dan is het voor mij ook makkelijk om te praten.”

10 Original in Dutch: “gestimuleerd om met te doen wat ik leuk vind.”

11 Original in Dutch: “rustgevende geluiden laten me in slaap vallen.”

12 Original in Dutch: “dat je leven om je heen hoort, maakt het aangenaam en gezellig. In dit geval maakt het hier erg gemoedelijk en gezellig.”

13 Original in Dutch: “muziek geeft sfeer, water er mensen ook. Tram verstoord beetje maar mocht erbij hier in de stad.”

14 Original in Dutch: “was teveel bezig met mijn eigen activiteit dat ik me weinig bewust was van het omgevings geluid.”

15 Original in Dutch: “rustige omgeving met een fontein en vogels.”

16 Original in Dutch: “veel mensen want het is mooi weer. Tram + auto ook verwacht omdat we vlakbij de weg zitten. Oosterpark is niet zo groot.”

17 Original in Dutch: “drukte, veel groepjes mensen, voetballende jongens.”

18 Original in Dutch: “een park, zoals elk ander park in A’dam.”

19 Original in Dutch: “ik ben hier vaker geweest dus wist wat ik kon verwachten.”
